# Sabemos prescrever profilaxia de tromboembolismo venoso nos pacientes internados?

**DOI:** 10.1590/1677-5449.008516

**Published:** 2017

**Authors:** Bruno Abdala Candido Lopes, Isabela Pizzatto Teixeira, Taynara Dantas de Souza, Jean Rodrigo Tafarel

**Affiliations:** 1 Pontifícia Universidade Católica do Paraná – PUCPR, Departamento de Clínica Médica, Curitiba, PR, Brasil.

**Keywords:** tromboembolismo venoso, profilaxia tromboembólica, embolia pulmonar

## Abstract

**Contexto:**

Embora preconizada, a profilaxia de tromboembolismo venoso (TEV) deixa de ser realizada sistematicamente em pacientes internados.

**Objetivo:**

Verificar se os pacientes hospitalizados recebem a prescrição correta da profilaxia de TEV do médico responsável por sua internação, conforme sua categoria de risco.

**Métodos:**

Estudo transversal com análise de prontuários de pacientes internados no Hospital Santa Casa de Misericórdia de Curitiba, PR, entre 20 de março e 25 de maio de 2015. Excluíram-se os pacientes em uso de anticoagulantes ou com sangramento ativo. Analisou-se gênero, idade, tipo de cobertura de saúde, especialidade responsável pelo paciente e fatores de risco dos pacientes para classificá-los em alto, moderado ou baixo risco para TEV. Comparou-se o uso ou não da profilaxia entre as prescrições das especialidades clínicas e cirúrgicas, pacientes internados pelo Sistema Único de Saúde (SUS) e por convênios e de acordo com seu risco para TEV.

**Resultados:**

Dos 78 pacientes avaliados, oito preencheram os critérios de exclusão. Dos 70 pacientes elegíveis (média etária 56,9 anos; 41 homens; 62 cobertos pelo SUS), 31 eram tratados por clínicos e 39 por cirurgiões. Apenas 46 (65,71%) pacientes receberam profilaxia para TEV. Dentre os pacientes clínicos, 29 (93,5%) receberam profilaxia, contra 17 (43,6%) do grupo cirúrgico (p < 0,001). Pacientes clínicos de moderado e alto risco receberam mais profilaxia que os cirúrgicos (p < 0,001 e p = 0,002). Não houve diferenças quanto à cobertura de saúde (SUS *versus* convênios médicos).

**Conclusões:**

No Hospital Santa Casa de Misericórdia de Curitiba, pacientes cirúrgicos estão menos protegidos de eventos tromboembólicos em relação aos clínicos.

## INTRODUÇÃO

O tromboembolismo venoso (TEV) é uma complicação comum em pacientes hospitalizados. Essa condição inclui a trombose venosa profunda (TVP) e o tromboembolismo pulmonar (TEP)^1^. Aproximadamente um terço dos pacientes hospitalizados possui risco de desenvolver TVP, número que pode ser reduzido substancialmente por meio da correta profilaxia^2^. O TEP é responsável por 5-10% das mortes em pacientes hospitalizados, fazendo com que o TEV seja a principal causa de morte evitável nesses pacientes^3^.

Sua incidência e recorrência nos Estados Unidos da América são estimadas em aproximadamente 900.000 casos por ano, com uma mortalidade estimada em 300.000, sendo que um terço evolui para morte súbita^4^. No Brasil, acredita-se que a prevalência chegue a 16,6%. Entretanto, os estudos da epidemiologia são escassos e acredita-se que a doença esteja subestimada devido aos eventos não diagnosticados^5^.

O estudo transversal ENDORSE, realizado em 358 hospitais distribuídos em 32 países, avaliou a prevalência de pacientes internados com risco para TEV e a proporção dos que receberam a correta profilaxia. Concluiu-se que aproximadamente metade desses pacientes receberam a profilaxia recomendada pelas diretrizes do American College of Chest Physicians (ACCP). Ou seja, mundialmente, a correta profilaxia para TEV é subutilizada^3^.

Além disso, a ocorrência do TEV traz impactos durante a hospitalização e no período pós-alta. Na internação, agrega maior morbimortalidade, aumento da permanência hospitalar e aumento dos gastos com saúde^1^^,^ 4 ^-^ 7. No pós-alta, nos pacientes que sobrevivem ao evento trombótico ou tromboembólico, podem-se encontrar em sua fase crônica casos de incapacitação física devido à evolução para síndrome pós-trombótica e insuficiência venosa crônica grave, que levam a um maior custo socioeconômico^1^^,^ 8.

Desta forma, devido ao impacto do TEV na evolução dos pacientes, sua profilaxia foi aprimorada e hoje está consolidada como um método efetivo para preveni-lo^9^. No entanto, a questão é se sabemos usá-la de forma correta e efetiva.

## MÉTODOS

Realizou-se um estudo transversal através da análise de prontuários médicos de pacientes internados no Hospital Santa Casa de Misericórdia de Curitiba, PR, no período compreendido entre 20 de março e 25 de maio de 2015.

Foram analisados dados relativos a prontuários médicos, mediante autorização concedida pelo diretor técnico do hospital e a garantia de sigilo em relação a todas as informações coletadas por parte dos pesquisadores, conforme o termo de compromisso de utilização de dados (TCUD). Esta pesquisa foi aprovada pelo Comitê de Ética em Pesquisa (CEP) da Pontifícia Universidade Católica do Paraná (PUCPR), Curitiba, PR, conforme consta no parecer 981.254 de 4 de março de 2015.

Os critérios de inclusão foram pacientes internados no Hospital Santa Casa de Misericórdia de Curitiba, PR, independente da especialidade prescritora, entre os dias 20 de março e 25 de maio de 2015. Os critérios de exclusão foram pacientes em uso de anticoagulantes orais e pacientes com sangramento ativo de qualquer localização. Os dados de cada paciente foram analisados uma única vez.

Em cada prontuário coletaram-se dados referentes a um único dia do atual internamento: cobertura de saúde [Sistema Único de Saúde (SUS) ou medicina privada], especialidade responsável pela prescrição do paciente (clínica ou cirúrgica, sendo considerado paciente cirúrgico aquele que possuía plano cirúrgico ou que havia sido submetido a algum procedimento cirúrgico), gênero, idade, classificação quanto ao risco para TEV e prescrição de profilaxia mecânica ou farmacológica para TEV. Os fatores de risco cirúrgicos e clínicos avaliados se encontram na Tabela 1.

**Tabela 1:** Fatores de risco para trombose venosa profunda^1^^,^ 9 ^,^ 10.

**Fatores clínicos**	**Fatores Cirúrgicos**	**Medicamentos**
Idade	Procedimento cirúrgico	Anticoncepcional oral
Neoplasia maligna	Presença de trauma	Terapia de reposição hormonal
Cateteres central e SG	Tipo de cirurgia	Quimioterapia
DII	Tipo de anestesia	Hormonioterapia
Doença respiratória grave	Grande queimado	Presença de infecção
Doença reumatológica	Imobilização	Insuficiência arterial ou venosa
Trombofilia	Grandes amputações	Internação em UTI
AVE	História prévia de TEV	Paresia ou paralisia de MMII
Gravidez	IAM	Síndrome nefrótica
Estado pós-parto	IC III ou IV de NYHA	Varizes

SG: Swan-Ganz; DII: doença inflamatória intestinal; AVE: acidente vascular encefálico; TEV: tromboembolismo endovenoso; IAM: infarto agudo do miocárdio; IC: insuficiência cardíaca; NYHA: New York Health Association; UTI: unidade de terapia intensiva; MMII: membros inferiores.

Cada paciente foi classificado quanto ao seu risco para TEV (baixo, moderado e alto), conforme Normas de Orientação Clínica para Prevenção, Diagnóstico e Tratamento da Trombose Venosa Profunda, da Sociedade Brasileira de Angiologia e Cirurgia Vascular (Tabelas 2 e 3)^10^. Conforme o risco atribuído a cada paciente, foi analisada a prescrição ou não da profilaxia. Naqueles que receberam profilaxia farmacológica, analisou-se se o fármaco e a dose prescrita estavam corretos. Considerou como prescrição correta de profilaxia para TEV para pacientes clínicos e cirúrgicos^1^^,^ 9 ^,^ 10:

**Tabela 2:** Avaliação do risco de trombose venosa profunda em doentes cirúrgicos^8^.

**Baixo risco**	**Médio risco**	**Alto risco**
Operações em pacientes de menos de 40 anos sem fatores de risco;	Cirurgia maior (geral, urológica ou ginecológica) em pacientes de 40 a 60 anos sem fatores adicionais de risco;	Cirurgia geral em paciente de mais de 60 anos;
Operações menores (de menos de 30 minutos e sem necessidade de repouso prolongado) em pacientes de mais de 40 anos sem outro fator de risco que não idade;	Cirurgia em pacientes de menos de 40 anos tomando estrógenos	Cirurgia geral em pacientes de 40-60 anos com fatores de risco adicionais;
Trauma menor		Cirurgia maior em pacientes com história de TVP ou TEP pregressa ou trombofilia;
		Grandes amputações;
		Cirurgias ortopédicas maiores;
		Cirurgias maiores em pacientes com neoplasias malignas;
		Cirurgias maiores em pacientes com outros estados de hipercoagulabilidade;
		Traumas múltiplos com fraturas de pélvis, quadril ou membros inferiores

TVP: trombose venosa profunda; TEP: tromboembolismo pulmonar.

**Tabela 3:** Avaliação do risco de trombose venosa profunda em doentes clínicos^7^.

**Baixo risco**	**Médio risco**	**Alto risco**
Qualquer doente.	Pacientes com mais de 65 anos acamados por doenças clínicas, sem outros fatores de risco.	Qualquer doença associada à TVP ou TEP prévia;
		Qualquer doença associada à trombofilia;
		Infarto do miocárdio;
		Doenças associadas a outros fatores de risco para TVP;
		Acidente vascular encefálico;
		Lesão de medula;
		Pacientes em UTI.

TVP: trombose venosa profunda; TEP: tromboembolismo pulmonar; UTI: unidade de terapia intensiva.

- Pacientes de baixo risco: movimentação no leito e deambulação precoce, descritas em prontuário;
- Pacientes de risco moderado: heparina não fracionada (HNF) na dose de 5.000 UI por via subcutânea a cada 12 horas, ou heparina de baixo peso molecular (HPBM) na dose de 20 mg por via subcutânea uma vez ao dia;
- Pacientes de alto risco: HNF 5000 UI por via subcutânea a cada 8 horas, ou HBPM na dose de 40 mg por via subcutânea uma vez ao dia.

Para fins de comparação, usou-se uma adaptação do Escore de Pádua para os doentes clínicos, para que pudessem ser classificados em baixo, moderado e alto risco (como os doentes cirúrgicos) e não apenas em pacientes com necessidade ou não de profilaxia. Esse escore foi utilizado por Engelhorn et al.^1^ no mesmo hospital escola, permitindo uma comparação entre os dados em nosso serviço, através de um escore utilizado previamente no estudo desses autores.

Os dados foram coletados e armazenados em uma planilha do Microsoft Excel. Os resultados obtidos de variáveis qualitativas foram descritos por frequências e percentuais. Variáveis quantitativas foram descritas por médias e desvios padrões. Para avaliar a associação entre duas variáveis qualitativas, foi usado o teste de qui-quadrado ou o teste exato de Fisher. Valores de p < 0,05 indicaram significância estatística. Os dados foram analisados com o programa computacional IBM SPSS Statistics v.20.0.

## RESULTADOS

Dos 78 prontuários analisados, oito pacientes foram excluídos, pois preenchiam os critérios de exclusão. Dos 70 pacientes incluídos no estudo, 41 (58,57%) pertenciam ao gênero masculino. A idade variou entre 17 e 91 anos (média etária de 56,9 anos) e 39 (55,71%) eram pacientes cirúrgicos. Quanto à cobertura de saúde durante a internação, 62 (88,57%) estavam internados pelo SUS, e 8 (11,43%) por algum convênio médico.

Do total da amostra coletada no Hospital Santa Casa de Misericórdia de Curitiba, 46 (65,71%) pacientes receberam profilaxia para TEV, enquanto 24 (34,29%) não a receberam. Comparando esses valores entre as especialidades clinicas e cirúrgicas, foi observado que, dos pacientes clínicos, 29 (93,5%) receberam profilaxia. Já entre os pacientes cirúrgicos, 17 (43,6%) receberam profilaxia para TEV e 22 (56,4%) não (p < 0,001; Tabela 4). Em relação à cobertura de saúde, 42 (67,7%) pacientes internados pelo SUS tiveram a profilaxia prescrita, contra apenas 4 (50%) do grupo pertencente aos convênios médicos (p = 0,432; Tabela 5).

**Tabela 4:** Prescrição de profilaxia para TEV entre pacientes clínicos e cirúrgicos.

**Prescrição da profilaxia**	**Pacientes clínicos**	**Pacientes cirúrgicos**
Profilaxia prescrita	30 (96,8%)	17 (43,6%)
Profilaxia não prescrita	1 (3,2%)	22 (56,4%)
Total	31	39

TEV: tromboembolismo venoso. p < 0,001.

**Tabela 5:** Prescrição de profilaxia para TEV entre pacientes do SUS e convênio.

**Prescrição da profilaxia**	**SUS**	**Convênio**
Profilaxia prescrita	42 (67,7%)	4 (50%)
Profilaxia não prescrita	20 (32,3%)	4 (50%)
Total	62	8

TEV: tromboembolismo venoso; SUS: Sistema Único de Saúde. p = 0,432.

No entanto, quanto à dose correta da profilaxia farmacológica para TEV, encontrou-se 24 (82,8%) prescrições corretas nos pacientes clínicos e 12 (70,6%), nos pacientes cirúrgicos (p = 0,462; Tabela 6).

**Tabela 6:** Utilização da profilaxia farmacológica na dose correta entre pacientes clínicos e cirúrgicos.

**Prescrição da profilaxia**	**Clínicos**	**Cirúrgicos**
Profilaxia prescrita na dose correta	24 (82,8%)	12 (70,6%)
Profilaxia prescrita na dose incorreta	5 (17,2%)	5 (29,4%)
Total	29	17

p = 0,462.

Correlacionou-se também se a profilaxia de TEV estava sendo prescrita com base no risco de TEV de cada paciente. Entre todos os pacientes de baixo risco (n = 8; 4 clínicos e 4 cirúrgicos), apenas 5 a receberam, com instruções de movimentação no leito e deambulação precoce descritas no prontuário, sendo 3 pacientes clínicos e 2 pacientes cirúrgicos (p = 1). Entre os pacientes de moderado risco (n = 23; 10 clínicos e 13 cirúrgicos), apenas 12 a receberam, sendo 10 pacientes clínicos e 2 pacientes cirúrgicos (p < 0,001). Já entre os pacientes de alto risco (n = 39; 17 clínicos e 22 cirúrgicos), 17 pacientes clínicos e 13 pacientes cirúrgicos receberam profilaxia (p = 0,002). Os resultados estão expostos nas Tabelas 7, 8 e 9.

**Tabela 7:** Profilaxia em pacientes de baixo risco.

**Prescrição da profilaxia**	**Clínicos**	**Cirúrgicos**
Profilaxia prescrita	3	2
Profilaxia não prescrita	1	2
Total	4	4

p = 1.

**Tabela 8:** Profilaxia em pacientes de moderado risco.

**Prescrição da profilaxia**	**Clínicos**	**Cirúrgicos**
Profilaxia prescrita	10	2
Profilaxia não prescrita	0	11
Total	10	13

p < 0,001.

**Tabela 9:** Profilaxia em pacientes de alto risco.

**Prescrição da profilaxia**	**Clínicos**	**Cirúrgicos**
Profilaxia prescrita	17	13
Profilaxia não prescrita	0	9
Total	17	22

p = 0,002.

O único esquema profilático medicamentoso empregado foi HPBM (enoxaparina). Não foi prescrito em nenhum prontuário o uso de métodos físicos – compressão pneumática intermitente e/ou meia elástica. Apenas um prontuário continha instruções de deambulação precoce.

## DISCUSSÃO

A profilaxia para fenômenos tromboembólicos possui benefício comprovado, além de ser mais oportuna em relação ao tratamento do TEV^11^. Todos os pacientes internados deveriam ser classificados quanto ao seu risco de desenvolver fenômenos tromboembólicos e receber a profilaxia adequada para preveni-los. Tanto a avaliação quanto a prescrição devem constar no prontuário médico^12^. Uma provável justificativa para a subutilização são as dúvidas quanto à classificação de risco e a indicação adequada para cada grupo^11^.

Os resultados do estudo demonstraram que 34,29% dos pacientes analisados não receberam profilaxia para TEV, apesar de haver indicação do benefício dessa profilaxia por diversos protocolos^6^^,^ 10. Vários estudos corroboram com nossos resultados que evidenciaram a subutilização da profilaxia de TEV na prática hospitalar 1 ^,^ 8 ^,^ 13 ^-^ 15. Pitta et al.^8^ observaram o não emprego da profilaxia em 83,5% dos pacientes em estudo realizado em 2006 no Hospital Escola Doutor José Carneiro em Maceió. Panju et al.^13^, em estudo realizado em dois hospitais acadêmicos canadenses, demonstraram que 54% dos pacientes que atendiam a critérios de inclusão receberam profilaxia para TEV. O estudo ENDORSE, de Cohen et al.^3^, que avaliou diferentes hospitais do mundo, mostrou que, dos pacientes internados, de 36% a 73% tinham risco para TEV, e a proporção dos pacientes que receberam a correta profilaxia variou entre 2% a 84%.

No presente estudo, foi demonstrada diferença estatística entre os pacientes clínicos e cirúrgicos. Entre os primeiros, 93,5% receberam alguma forma de prevenção, comparados aos 43,6% do total de pacientes cirúrgicos, evidenciando que pacientes clínicos estão mais protegidos que os pacientes cirúrgicos quanto ao risco de desenvolver TEV em nosso hospital. Da mesma forma, num estudo realizado por Garcia et al.^16^ no Centro Hospitalar Unimed de Joinville, mais de 2/3 dos pacientes com indicação não receberam profilaxia, e a maior omissão ocorreu nos pacientes cirúrgicos de médio risco. Tal fato pode estar relacionado com a preocupação equívoca por parte do cirurgião quanto ao risco de sangramento associado aos medicamentos utilizados na profilaxia, bem como à ausência de equipe hospitalar de vigilância para checar se a profilaxia foi instituída.

Em relação ao grupo de risco para TEV, observamos também que o grupo de pacientes de médio e alto risco tratados por clínicos receberam mais profilaxia que aqueles manejados por cirurgiões. Uma provável justificativa para a subutilização são novamente as dúvidas quanto à classificação de risco e à indicação adequada para cada grupo^4^^,^ 9 ^,^ 11. Esse ponto poderia ser resolvido com a criação de protocolo interno hospitalar e sua divulgação a todos os médicos do corpo clínico. Complementarmente, seria necessária a integração multidisciplinar de equipes e da direção hospitalar.

Em estudo realizado por Engelhorn et al.^1^, também no Hospital Santa Casa de Misericórdia de Curitiba, no ano de 2001, 87,28% do total de pacientes analisados não recebeu nenhuma forma de profilaxia de TEV. Comparando esses resultados com os do nosso estudo, podemos dizer que, nesse hospital, houve uma melhora da prescrição da profilaxia de TEV após 14 anos. Entretanto, naquele estudo não houve diferença significativa entre pacientes clínicos e cirúrgicos, fato que talvez possa ser atribuído ao caráter prospectivo do estudo.

Vale ressaltar o fato de que nenhum dos pacientes com sangramento ativo analisados em nosso estudo recebeu a profilaxia mecânica com compressão pneumática intermitente ou meias elásticas compressivas, o que é preconizado de acordo com as Normas de Orientação Clínica para a Prevenção, Diagnóstico e o Tratamento da Trombose Venosa Profunda^10^.

Podemos concluir que as medidas para a prevenção do TEV estão sendo subempregadas em nosso serviço, assim como em outros hospitais do Brasil^1^^,^ 8 ^,^ 15. No hospital escola analisado, também encontramos uma diferença significativa entre a realização de profilaxia correta entre pacientes clínicos e cirúrgicos.

É importante que se estimule a replicação de estudos como este em outros centros hospitalares, para incluir um número maior de pacientes. A validação dos resultados aqui apresentados pode expor com mais clareza as falhas do processo de prevenção do TEV entre clínicos e cirurgiões. Dessa forma, busca-se destacar a importância da necessidade de criação de projetos que facilitem o uso global das diversas formas de profilaxia para essa doença, visando combater sua alta morbimortalidade e diminuir os custos gerados por essa enfermidade.

## References

[1] Engelhorn ALV, Garcia ACF, Cassou MF, Bircholz L, Engelhorn CA (2002). Profilaxia da Trombose Venosa Profunda - estudo epidemiológico em um hospital escola. J Vasc Bras.

[2] Anderson FA, Zayaruzny M, Heit JA, Fidan D, Cohen AT (2007). Estimated annual numbers of US acute-care hospital patients at risk for venous thromboembolism. Am J Hematol.

[3] Cohen AT, Tapson VF, Bergmann JF (2008). Venous thromboembolism risk and prophylaxis in the acute hospital care setting ENDORSE study: a multinational cross-sectional study. Lancet.

[4] Caprini JA (2010). Risk assessment as a guide for the prevention of the many faces of venous thromboembolism. Am J Surg.

[5] Terra M, Menna Barreto SS, Rocha AT (2010). Recomendações para o Manejo da tromboembolia pulmonar. J Bras Pneumol.

[6] Qaseem A, Chou R, Humphrey LL, Starkey M, Shekelle P (2014). Venous thromboembolism prophylaxis in hospitalized patients: a clinical practice guideline from the American College of Physicians. Ann Intern Med.

[7] Cardoso LF (2015). Protocolo de Profilaxia de Tromboembolismo Venoso em Pacientes Internados.

[8] Pitta GBB, Leite TL, Silva MDC, Melo CFL, Calheiros GA. (2007). Avaliação da utilização de profilaxia da trombose venosa profunda em um hospital escola. J Vasc Bras.

[9] Barbar S, Noventa F, Rossetto V (2010). A risk assessment model for the identification of hospitalized medical patients at risk for venous thromboembolism: the Padua Prediction Score. J Thromb Haemost.

[10] Maffei FHA, Caiafa JS, Ramacciotti E (2005). Normas de orientação clínica para prevenção, diagnóstico e tratamento da trombose venosa profunda. J Vasc Bras.

[11] Nicolaides AN, Breddin HK, Fareed J (2001). Prevention of venous thromboembolism. International Consensus Statement. Guidelines compiled in accordance with the scientific evidence. Int Angiol.

[12] Garcia G, Peret F, Maciel R (2013). Protocolos Clínicos 045: Prevenção de Tromboembolia Venosa.

[13] Panju M, Raso D, Patel A, Panju A, Ginsberg J (2011). Evaluation of the use of venous thromboembolism prophylaxis in hospitalised medical patients. J R Coll Physicians Edinb.

[14] Dentali F, Douketis JD, Gianni M, Lim W, Crowther MA (2007). Meta-analysis: Anticoagulant prophylaxis to prevent symptomatic venous thromboembolism in hospitalized medical patients. Ann Intern Med.

[15] Franco RM, Simezo V, Bortoleti RR (2006). Profilaxia para tromboembolismo venoso em um hospital de ensino. J Vasc Bras.

[16] Garcia ACF, Souza BV, Volpato DE (2005). Realidade do uso da profilaxia para trombose venosa profunda: da teoria à prática. J Vasc Bras.

