# Supplementary material for: Sabemos prescrever profilaxia de tromboembolismo venoso nos pacientes internados?
Source: J Vasc Bras. 2017 Jul-Sep;16(3):199–204. [Article in Portuguese] doi: 10.1590/1677-5449.008516 (PMC5868935; doi:10.1590/1677-5449.008516)
Supplement: Translation

# Translation: Do we know how to prescribe venous thromboembolism prophylaxis to hospitalized patients?

**Authors:** Bruno Abdala Candido Lopes, Isabela Pizzatto Teixeira, Taynara Dantas de Souza, Jean Rodrigo Tafarel

**Affiliations:** 1 Pontifícia Universidade Católica do Paraná – PUCPR, Departamento de Clínica Médica, Curitiba, PR, Brazil.

**Keywords:** venous thromboembolism, thromboembolic prophylaxis, pulmonary embolism

## Abstract

**Background:**

Although prophylaxis to prevent venous thromboembolism is recommended, it is rarely systematically performed in hospitalized patients.

**Objective:**

To investigate whether hospitalized patients are given the correct VTE prophylaxis prescription by the physician responsible for them while in hospital, analyzed by risk category.

**Methods:**

This was a cross-sectional study based on analysis of medical records for patients admitted to the Hospital Santa Casa de Misericórdia, Curitiba, PR, Brazil, from March 20 to May 25, 2015. Patients on anticoagulants or with active bleeding were excluded. The following variables were analyzed: sex, age, type of healthcare coverage, specialty responsible for the patient, and patients’ risk factors to classify them as at high, moderate, or low risk of VTE. Use or not of prophylaxis was compared across prescriptions made by clinical and surgical specialties, between patients treated on the Brazilian National Health Service (SUS - Sistema Único de Saúde) and private health insurance, and according to patients’ risk of VTE.

**Results:**

Eight of the 78 patients assessed met exclusion criteria. The remaining 70 eligible patients had a mean age of 56.9 years, 41 were male, 62 were treated on the SUS, 31 were treated by clinicians, and 39 were treated by surgeons. Just 46 (65.71%) patients were given prophylaxis for VTE. Among the clinical patients, 29 (93.5%) were given prophylaxis, against 17 (43.6%) in the surgical group (p < 0.001). Moderate and high risk clinical patients were more likely to be given prophylaxis than surgical patients (p < 0.001 and p = 0.002). There were no differences with relation to type of healthcare coverage (SUS vs. private healthcare).

**Conclusions:**

At the Hospital Santa Casa de Misericórdia in Curitiba, surgical patients are less well protected from thromboembolic events than clinical patients.

## INTRODUCTION

Venous thromboembolism (VTE) is a common complication among hospitalized patients. The condition includes deep venous thrombosis (DVT) and pulmonary thromboembolism (PTE).^1^ Approximately one third of hospitalized patients are at some risk of developing DVT, but this number can be reduced substantially by correct prophylaxis.^2^ Around 5-10% of deaths among hospitalized patients are caused by PTE, with the result that VTE is the number one cause of avoidable deaths among these patients.^3^

In the United States, its incidence and recurrence are estimated at approximately 900,000 cases per year, with an estimated mortality of 300,000, with a third progressing to sudden death.^4^ In Brazil, it is estimated that prevalence reaches 16.6%. However, epidemiological studies are scarce and it is believed that the disease is underestimated because of undiagnosed events.^5^

The ENDORSE cross-sectional study was conducted at 358 hospitals distributed across 32 countries to assess the prevalence of hospitalized patients at risk of VTE and the proportion of them who were given the correct prophylaxis. It was concluded that approximately half of those patients were given the prophylaxis recommended by the American College of Chest Physicians (ACCP) guidelines. The correct prophylaxis for VTE is underutilized globally.^3^

Additionally, the impact of VTE extends beyond the hospital stay into the post-discharge period. In hospital, it increases morbidity and mortality, extends the length of hospital stays, and increases healthcare spending.^1^^,^ 4 ^-^ 7 After discharge, patients who survive a thrombotic or thromboembolic event can face physical incapacity during the chronic phase, caused by progression to postthrombotic syndrome and severe chronic venous insufficiency, increasing the socioeconomic burden.^1^^,^ 8

The severity of VTE’s impact on patients prompted improvements to prophylaxis and it is now consolidated as an effective method for preventing these events.^9^ However, the question is whether we know how to use it correctly and effectively.

## METHODS

A cross-sectional study was conducted on the basis of analysis of medical records for patients admitted to the Hospital Santa Casa de Misericórdia, Curitiba, PR, Brazil, between March 20 and May 25, 2015.

Data from medical records were analyzed, with authorization from the technical director of the hospital and confidentiality of all information collected was guaranteed by the researchers, who signed a data usage contract. The study was approved by the Research Ethics Committee at the Pontifícia Universidade Católica do Paraná (PUCPR), Curitiba, PR, Brazil, as recorded in ruling 981.254 of March 4, 2015.

Inclusion criteria were patients admitted to the Hospital Santa Casa de Misericórdia in Curitiba, irrespective of the treating specialty, between March 20 and May 25, 2015. Exclusion criteria were patients taking oral anticoagulants and patients with active bleeding at any site. Each patient’s data were only included in the analysis once.

From each medical record, data were collected for a single day in hospital on the following variables: healthcare cover (Brazilian National Health Service [SUS] or private medicine), specialty responsible for patient’s prescriptions (clinical or surgical, where patients were considered surgical if there was surgery planned or if they had undergone a surgical procedure), sex, age, classification of VTE risk, and prescription of mechanical or pharmacological prophylaxis for VTE. The surgical and clinical risk factors analyzed are shown in Table 1.

**Table 1:** Risk factors for deep venous thrombosis.^1^^,^ 9 ^,^ 10

**Clinical factors**	**Surgical factors**	**Medications**
Age	Surgical procedure	Oral contraception
Malignant neoplasm	Trauma	Hormone replacement therapy
Central and SG catheters	Type of surgery	Chemotherapy
IID	Type of anesthesia	Hormone therapy
Severe respiratory disease	Major burns	Infection
Rheumatological disease	Immobilization	Arterial or venous insufficiency
Thrombophilia	Major amputations	ICU admission
Stroke	Prior history of VTE	LL paresis or paralysis
Pregnancy	AMI	Nephrotic syndrome
Postpartum status	NYHA class III or IV HF	Varicose veins

SG: Swan-Ganz; IID: inflammatory intestinal disease; VTE: venous thromboembolism; AMI: acute myocardial infarction; NYHA: New York Health Association; HF: heart failure; ICU: intensive care unit; LL: lower limbs.

Each patient was classified for VTE risk (low, moderate, or high), according to clinical guidelines for prevention, diagnosis and treatment of deep venous thrombosis published by the Brazilian Society of Angiology and Vascular Surgery (Tables 2 and 3).^10^ Prophylaxis prescription or non-prescription was analyzed patient by patient according to the risk attributed to each. For patients prescribed pharmacological prophylaxis, the drugs chosen and dosage administered were also analyzed. The following prescriptions of VTE prophylaxis methods for clinical and surgical patients were defined as correct:^1^^,^ 9 ^,^ 10

**Table 2:** Assessment of risk of deep venous thrombosis in surgical patients.^8^

**Low risk**	**Moderate risk**	**High risk**
Operations on patients less than 40 years old without risk factors;	Major surgery (general, urological, or gynecological) in patients aged 40 to 60 years, without additional risk factors;	General surgery in patients over 60 years old;
Minor operations (duration of less than 30 minutes and with no need for prolonged rest) in patients over 40 years old without additional risk factors other than age;	Surgery in patients less than 40 years old who are taking estrogens	General surgery in patients aged 40-60 years with additional risk factors;
Minor trauma		Major surgery in patients with a previous history of DVT or PTE or thrombophilia;
		Major amputations;
		Major orthopedic surgery;
		Major surgery in patients with malignant neoplasms;
		Major surgery in patients with other hypercoagulable states;
		Multiple traumas with fractures of pelvis, hips, or lower limbs

DVT: deep venous thrombosis; PTE: pulmonary thromboembolism.

**Table 3:** Assessment of risk of deep venous thrombosis in clinical patients.^7^

**Low risk**	**Moderate risk**	**High risk**
Any patient.	Patients over the age of 65 years, bedridden by clinical diseases, but with no other risk factors.	Any disease combined with previous DVT or PTE; Any disease combined with thrombophilia; Myocardial infarction; Diseases associated with other risk factors for DVT; Stroke; Spinal cord injury; Patients in ICU.

DVT: deep venous thrombosis; PTE: pulmonary thromboembolism; ICU: intensive care unit.

- Low risk patients: movement in bed and early mobilization, described in the patient’s record;
- Moderate risk patients: subcutaneous unfractionated heparin (UFH) at a dosage of 5,000 UI every 12 hours, or 20 mg of subcutaneous low molecular weight heparin (LMWH), once a day;
- High risk patients: 5000 UI of subcutaneous UFH every 8 hours, or 40 mg of subcutaneous LMWH once a day.

For the purposes of comparison, an adapted version of the Pádua score was used for clinical patients to enable their classification as low, moderate, or high risk (in common with surgical patients) rather than as patients who did or did not need prophylaxis. This score has been used previously by Engelhorn et al.^1^ at the same teaching hospital, enabling comparison with previous data for our service, using a score already applied in their study.

Data were collected and stored in a Microsoft Excel spreadsheet. The results for qualitative variables were expressed as frequencies and percentages. Quantitative variables were expressed as means and standard deviations. Associations between two qualitative variables were evaluated using the chi-square test or Fisher’s exact test. Values of p < 0.05 indicate statistical significance. Data were analyzed using IBM SPSS Statistics v.20.0.

## RESULTS

A total of 78 patient medical records were analyzed, from which eight patients were excluded because they met the exclusion criteria. Of the remaining 70 patients included in the study, 41 (58.57%) were male. Ages ranged from 17 to 91 years (mean age of 56.9 years) and 39 (55.71%) were surgical patients. With regard to healthcare cover, 62 (88.57%) were admitted on the SUS, and 8 (11.43%) by a private health insurer.

In the entire sample of patients admitted to the Hospital Santa Casa de Misericórdia in Curitiba, 46 (65.71%) patients were given VTE prophylaxis and 24 (34.29%) were not. Analyzing the data for clinical versus surgical specialties reveals that 29 clinical patients (93.5%) were given prophylaxis, whereas 17 surgical patients (43.6%) were given VTE prophylaxis and 22 (56.4%) were not (p < 0.001; Table 4). With relation to healthcare coverage, 42 (67.7%) patients admitted on the SUS had prophylaxis prescribed, compared to just 4 (50%) patients admitted via health insurance (p = 0.432; Table 5).

**Table 4:** Prescription of prophylaxis for VTE in clinical and surgical patients.

**Prescription of prophylaxis**	**Clinical patients**	**Surgical patients**
Prophylaxis prescribed	30 (96.8%)	17 (43.6%)
Prophylaxis not prescribed	1 (3.2%)	22 (56.4%)
Total	31	39

VTE: venous thromboembolism. p < 0.001.

**Table 5:** Prescription of prophylaxis for VTE in patients treated on SUS and privately.

**Prescription of prophylaxis**	**SUS**	**Private**
Prophylaxis prescribed	42 (67.7%)	4 (50%)
Prophylaxis not prescribed	20 (32.3%)	4 (50%)
Total	62	8

VTE: venous thromboembolism; SUS: Brazilian National Health Service (Sistema Único de Saúde). p = 0.432.

However, correct pharmacological VTE prophylaxis was observed in 24 (82.8%) clinical patients and 12 (70.6%) surgical patients (p = 0.462; Table 6).

**Table 6:** Use of correct dosage of pharmacological prophylaxis in clinical and surgical patients.

**Prescription of prophylaxis**	**Clinical**	**Surgical**
Prophylaxis prescribed at correct dosage	24 (82.8%)	12 (70.6%)
Prophylaxis prescribed at incorrect dosage	5 (17.2%)	5 (29.4%)
Total	29	17

p = 0.462.

VTE prophylaxis prescriptions was also analyzed on the basis of each patient’s risk of VTE. Just 5 (3 clinical patients and 2 surgical patients, p = 1) of the low VTE risk patients (n = 8; 4 clinical and 4 surgical patients) were prescribed prophylaxis, with instructions for movement in bed and early mobilization described in their patient records. Just 12 patients (10 clinical and 2 surgical, p < 0.001) with moderate VTE risk (n = 23; 10 clinical and 13 surgical patients) were given prophylaxis. Among the high VTE risk patients (n = 39; 17 clinical and 22 surgical patients), 17 clinical patients and 13 surgical patients were given prophylaxis (p = 0.002). These results are shown in Tables 7, 8, and 9.

**Table 7:** Prophylaxis in low risk patients.

**Prescription of prophylaxis**	**Clinical**	**Surgical**	
Prophylaxis prescribed	3		2
Prophylaxis not prescribed	1		2
Total	4		4

p = 1.

**Table 8:** Prophylaxis in moderate risk patients.

**Prescription of prophylaxis**	**Clinical**	**Surgical**
Prophylaxis prescribed	10	2
Prophylaxis not prescribed	0	11
Total	10	13

p < 0.001.

**Table 9:** Prophylaxis in high risk patients.

**Prescription of prophylaxis**	**Clinical**	**Surgical**
Prophylaxis prescribed	17	13
Prophylaxis not prescribed	0	9
Total	17	22

p = 0.002.

The only drug used for prophylaxis was a LMWH (enoxaparin). None of the patient records mentioned use of physical methods – intermittent pneumatic compression and/or elastic stockings. Just one patient record contained instructions for early mobilization.

## DISCUSSION

The benefits of prophylaxis against thromboembolic phenomena have been proven and it is more opportune than treating VTE.^11^ All hospitalized patients should be classified in terms of their risk of developing thromboembolic phenomena and should be given the correct prophylaxis to prevent it. Both the assessment and the prescription should be included in the patient’s medical record.^12^ One possible explanation for underutilization is doubts with relation to risk classification and the correct prophylaxis for each group.^11^

The results of the study demonstrated that 34.29% of the patients analyzed were not given VTE prophylaxis, despite the benefits of such prophylaxis being clearly stated in many different protocols.^6^^,^ 10 Several other studies have reported results in line with ours, showing underutilization of VTE prophylaxis in hospital practice.^1^^,^ 8 ^,^ 13 ^-^ 15 Pitta et al.^8^ observed that prophylaxis was not employed in 83.5% of the patients in a study conducted in 2006 at the Hospital Escola Doutor José Carneiro, in Maceió, Brazil. Panju et al.^13^ conducted a study at two teaching hospitals in Canada and showed that 54% of the patients who met inclusion criteria received VTE prophylaxis. The ENDORSE study, by Cohen et al.,^3^ assessed many different hospitals globally and showed that 36% to 73% of patients in hospital were at risk of VTE, while the proportion of patients who were given the correct prophylaxis varied from 2% to 84%.

The present study found a statistical difference between clinical and surgical patients. Among the clinical patients, 93.5% received some form of prevention, compared to 43.6% of the total number of surgical patients, showing that at our hospital clinical patients are better protected from the risk of developing VTE than surgical patients. Similarly, a study conducted by Garcia et al.,^16^ at the Centro Hospitalar Unimed, in Joinville, Brazil, found that more than 2/3 of patients with indications were not given prophylaxis, and the greatest rate of omission was observed among moderate risk surgical patients. This could be related to a unfounded concern on the part of the surgeon with relation to the risk of bleeding associated with medications used for prophylaxis, and also to the lack of a hospital surveillance team to verify whether prophylaxis is employed.

With relation to the group of patients at risk of VTE, we also observed that the moderate and high risk groups treated by clinicians were more likely to receive prophylaxis than those managed by surgeons. Once more, the probable explanation for underutilization is doubts with regard to risk classification and the correct indications for each group.^4^^,^ 9 ^,^ 11 This could be resolved by creating an internal hospital protocol and making all physicians in the clinical staff aware of it. This would need to be supplemented by multidisciplinary integration of teams and the hospital management.

A study conducted by Engelhorn et al.,^1^ also at the Hospital Santa Casa de Misericórdia in Curitiba, in 2001, found that 87.28% of all of the patients analyzed were not given any form of VTE prophylaxis. Comparing those results with the results of our study, we can state that there has been an improvement in VTE prophylaxis prescription over the 14 years that have elapsed since then. However, in that study there was no significant difference between clinical and surgical patients, which could possibly be attributed to the prospective study design.

It should also be pointed out that none of the patients with active bleeding analyzed in our study were given mechanical prophylaxis with intermittent pneumatic compression or elastic compression stockings, as recommended by the clinical guidelines for prevention, diagnosis, and treatment of deep venous thrombosis.^10^

It can be concluded that measures for the prevention of VTE are being underutilized at our service, as is the case in other hospitals in Brazil.^1^^,^ 8 ^,^ 15 In the teaching hospital analyzed, we also found a significant difference between clinical and surgical patients in terms of the rates of correct prophylaxis.

It is important to encourage replication of studies such as this one at other hospitals, to include larger numbers of patients. Validation of the results presented here could more clearly expose the failures in VTE prevention processes among clinicians and surgeons. It is also important to highlight the need to set up projects that facilitate universal use of the many different forms of prophylaxis against this disease, with the objective of combating its high morbidity and mortality and reducing the costs generated.
